# Efficacy and safety of proprotein convertase subtilisin-kexin type 9 (PCSK9) inhibitors, alirocumab and evolocumab, a post-commercialization study

**DOI:** 10.1186/s12944-017-0493-7

**Published:** 2017-07-24

**Authors:** Joshua Choi, Amir M Khan, Michael Jarmin, Naila Goldenberg, Charles J Glueck, Ping Wang

**Affiliations:** Graduate Medical Education and Research, The Jewish Hospital- Mercy Health, Graduate Medical Education and Research, Cincinnati, USA

**Keywords:** PCSK9 inhibitor, Efficacy, Safety, Cardiovascular risk, Alirocumab, Evolocumab, Hypercholesterolemia, Low-density lipoprotein

## Abstract

**Background:**

Efficacy-safety of proprotein convertase subtilisin-kexin type 9 (PCSK9) inhibitors, alirocumab (ALI) and evolocumab (EVO), have previously been evaluated through controlled clinical trials with selective patient groups. Post-commercially, in 69 patients with heterozygous familial hypercholesterolemia (HeFH) and/or cardiovascular disease (CVD) with suboptimal LDL cholesterol (LDLC) lowering on maximal tolerated LDLC therapy, we assessed efficacy and safety of ALI and EVO.

**Methods:**

Post-commercially, we started 29 patients on ALI 75 mg, 18 on ALI 150 mg, and 22 on EVO 140 mg every 2 weeks added to a maximally tolerated LDLC-lowering regimen. Since LDLC lowering did not differ between ALI 150 and EVO 140 mg, ALI 150-EVO 140 data were pooled (ALI-EVO). Changes in LDLC and AHA and NIH calculated 10-year CVD risks were assessed.

**Results:**

Of the 69 patients, 25 had HeFH, 25 CVD, and 19 had both. At entry, 23 (33%) took statins and 46 (67%) were statin-intolerant. Mean ± SD and median follow-up were 49 ± 13 and 49 weeks on ALI 75 mg, and 37 ± 12 and 33 weeks on ALI-EVO. In the ALI-EVO group (*n* = 40), median LDLC fell from 165 mg/dl at entry to 70 mg/dl (median − 59%, *p* < .0001). AHA 10-year calculated CVD risk fell from 10.2 to 5.5% (median − 28%, *p* < .0001), and by the NIH calculator from 14.2 to 3.6% (median − 78%, *p* < .0001). In the ALI 75 mg group (*n* = 29), entry LDLC fell from 115 to 68 mg/dl (median − 39%, *p* < .0001). AHA 10-year calculated CVD risk fell from 11.5 to 7.3% (median − 20%, *p* = .004), and NIH 10-year risk from 12.9 to 5.1% (median 67%, *p* < .0001). Absolute and percent change in LDLC was independent of statin use. There were flu-like symptoms in 14% of patients. Adverse events did not differ (*p* > 0.05) between ALI 75 mg and ALI-EVO.

**Conclusion:**

In patients with HeFH and/or CVD, LDLC decreased from 115 to 68 mg/dl (39%) on ALI 75 mg with mean follow-up of 49 weeks, and from 165 to 70 mg/dl (59%) on ALI-EVO over 37 weeks, *p* < .0001 for both. Adverse events were minimal and tolerable. ALI and EVO represent paradigm shifts in LDLC lowering.

## Background

Before commercialization, efficacy and safety of alirocumab (ALI) and evolocumab (EVO) in patients has been evaluated through randomized controlled clinical trials [1–5] with stringent inclusion and exclusion criteria, creating highly selective cohorts of study patients. ALI and EVO have approved indications in patients with heterozygous familial hypercholesterolemia (HeFH), Simon Broom’s Criteria, [6] and/or WHO Dutch Lipid Criteria, [7] and/or in patients with cardiovascular disease (CVD) with suboptimal LDLC lowering despite maximal tolerated cholesterol lowering therapy. Previously, we have projected that an estimated 24 million Americans could be eligible for PCSK9 inhibitor therapy [8, 9].

ALI ODYSSEY Phase III studies demonstrated that the mean percentage change in calculated low-density lipoprotein cholesterol (LDLC) from baseline to week 24 beyond statin effect was −61% versus 0.8% (placebo), *p* < 0.001. [2, 10] The Odyssey Combo 1 phase 3 study enrolled 316 patients with CHD or CHD risk equivalents and hypercholesterolemia, providing ALI 75 mg every 2 weeks (Q2W) which was increased to 150 mg Q2W if week 8 LDLC was ≥70 mg/dl [11]. All patients also took maximally tolerated statin therapy. At week 24 mean LDL had fallen 46% beyond placebo, *p* < .0001. LDLC <70 was achieved by 75% on ALI vs 9% placebo at week 24. Treatment Emergent Adverse Events (TEAE) were comparable between groups.

The Odyssey Combo II trial [12] was a 104 week study of ALI 75 mg Q2W vs ezetimibe in patients with high cardiovascular risk and elevated LDLC despite maximal doses of statin. At week 24, mean reductions in LDLC were 50.6% for ALI vs 20.7% for ezetimibe, and 45.6% of patients achieved LDLC <70 mg/dl. There was no excess of TEAE when compared to ezetimibe.

In Odyssey Choice II, ALI 150 mg Q4W or 75 mg Q2W were used with dose adjustment to 150 Q2W at week 12 if pre-defined LDLC levels were not met [13]. Overall, 63.9% and 70.3% of ALI treated patients achieved their LDLC goals at week 24. Patients with inadequately controlled hypercholesterolemia and not on a statin were included.

In pooled analyses of 6 trials of ALI including 4211 patients for 52 weeks or longer, there was no evidence for transition to new-onset diabetes in 3448 cases without diabetes at entry with a follow up period of 6–18 months, compared to either placebo or ezetimibe [14]. The safety of ALI has been evaluated in pooled data from 14 trials, double blind treatment for 8 to 104 weeks, in 3340 ALI and 1894 controls (placebo or ezetimibe) with focus on patients with at least 2 consecutive LDLC levels <25 or once <15 mg/dl [15]. There was no increase in overall TEAE event rates or neurocognitive events, although cataract incidence appeared to be increased in the group achieving LDLC <25 mg/dl.

Sabatine et al. have reported that EVO reduced the level of LDLC by 61% from a median of 120 to 48 mg/dl. The rate of cardiovascular events at 1 year was reduced from 2.18% in the standard therapy group to 0.95% in the EVO group, hazard ratio 0.47, 95% CI 0.2–0.78, *p* = .003 [4]. In the LAPLACE-2 randomized trial, Robinson et al. reported that EVO reduced LDLC by 66 to 75% and by 63 to 75% vs placebo at the mean of weeks 10 and 12, respectively, in the moderate and high intensity statin-treated groups [16]. Nissen has reported efficacy and tolerance in patients with statin intolerance [1] with a 54% lowering of LDLC on EVO. Koren et al. [17] have recently summarized data from 4641 patient-years among 1255 patients randomized to EVO in one of five placebo-controlled phase II studies (GAUSS-1, RUTHERFORD-1, YUKAWA-1, MENDEL-1, or LAPLACE-TIMI 57), and followed-up for an average of 44 months. The median LDLC reduction over baseline was 57%, similar to the 61% seen at 12 months in the original studies. Safety was maintained in the open-label follow-up.

While OSLER-1 follow-up showed a median LDL of 60 mg/dL on long-term EVO, the second study -- a prespecified analysis of the intensive lipid-lowering IMPROVE-IT trial -- looked at the safety of levels below 30 mg/dL. Giugliano et al. [18] have recently reported “…patients achieving an LDL-C level less than 30 mg/dL at 1 month had a similar safety profile (and numerically the lowest rate of cardiovascular events) over a 6-year period compared with patients achieving higher LDL-C concentrations.”

In the Fourier trial, 27,564 patients with ASCVD and LDLC ≥70 mg/dl who were receiving statin therapy took EVO 140 mg Q2W or 420 mg every month with matching placebo, with median duration of follow-up 2.2 years [19]. The primary efficacy end point was the composite of cardiovascular death, myocardial infarction, stroke, hospitalization for unstable angina, or coronary revascularization. The key secondary efficacy end point was the composite of CVD death, MI, or stroke. At 48 weeks, LDLC on EVO was reduced 59% from 92 to 30 mg/dl. Relative to placebo, EVO reduced the risk of the primary endpoint 9.8% vs 11.3%, hazard ratio 0.85, 95% CI 0.79 to 0.92, *p* < .001 and the key secondary endpoint from 5.9% vs 7.4%, hazard ratio 0.80, 95% CI 0.73 to 0.89, *p* < .001. There were no case-control differences in adverse events, excepting injection site reactions, 2.1% with EVO vs 1.6% control [17, 19].

In the GLAGOV randomized trial [20] in 846 patients with evaluable imaging (IVUS) at follow-up, compared to placebo the EVO group had lower mean LDLC, 37 vs 93 mg/dl. EVO induced plaque regression in a greater percentage of patients than placebo, 64.3% vs 47.3%, difference 17%, 95% CI 10.4% to 23.6%, *p* = .001. After 76 weeks of treatment, EVO compared to placebo, resulted in a greater decrease in PAV.

We previously carried out an open label efficacy and safety 24-week study of ALI and EVO in 72 patients with HeFH and/or CVD with suboptimal LDL cholesterol (LDLC) lowering on maximal tolerated cholesterol lowering therapy [21]. Post-commercially, we started 25 patients on ALI 75 mg, 15 on ALI 150 mg, and 32 on EVO 140 mg every 2 weeks added to a maximally tolerated entry LDLC lowering regimen, with follow-up for a median 24 weeks. At 24 weeks, on ALI 75 mg, median LDLC decreased from 117 to 62 mg/dL (−54%), on ALI 150 mg, LDLC fell from 175 to 57 mg/dL (−63%), and on EVO 140 mg, LDLC fell from 165 to 69 mg/dL (−63%), *p* < 0.0001 for all. Absolute and percent LDLC reduction did not differ (*p* > .05) between ALI 150 and EVO 140 mg, but were less on ALI 75 mg vs ALI 150 mg and EVO 140 mg (*p* < .05) [21]. Percent reductions in 10-year CVD risks by AHA and NIH calculators, respectively were ALI 75 mg −22% and −44%, ALI 150 mg −31% and −50%, and EVO 140 mg −29% and −56%, *p* ≤. 002 for all [21]. The three most common adverse events included flu-like myositis 10%, respiratory tract symptoms 8%, and injection site reaction 6% [21]. Adverse events were minimal and tolerable.

Statin intolerance, predominantly myalgia, myositis, and myopathy, occurs in 10–29% of statin-treated patients [22, 23]. In the GAUSS-3 study of patients with previous statin intolerance, 43% of patients on atorvastatin had muscular symptoms. When ezetimibe and placebo were compared to EVO and placebo, 29% experienced myalgias on ezetimibe versus 21% of those on EVO [1]. Furthermore, LDLC reduction from baseline on ezetimibe was −17% versus −53% on EVO at 24 weeks. In these patients with statin intolerance, EVO was effective and well-tolerated [1].

Our specific aim, in an extended [21] post-commercialization, open label study, was to assess the safety and efficacy of ALI and EVO in lowering LDLC, and subsequent change in calculated 10-year CVD risk in patients with HeFH and/or CVD referred to a regional cholesterol center for diagnosis and treatment of hypercholesterolemia.

## Methods

The procedures were in accordance with the ethical standards of human experimentation, and approved by The Jewish Hospital Institutional Review Board.

Since the commercialization of PCSK9 inhibitors in July 2015 at our regional cholesterol center, 69 patients had extended (>24 weeks) follow up on either EVO 140 mg Q2W (*n* = 22) or ALI 150 mg Q2W (*n* = 18) or ALI 75 Q2W (*n* = 29). They qualified for PCSK9 therapy by HeFH (Simon Broom’s Criteria [6], WHO Dutch Lipid Criteria score > 8 [7]), and/or CVD with suboptimal LDLC lowering despite maximal tolerated cholesterol lowering therapy, including statin doses down to zero. HeFH was assessed by the presence of tendon xanthomas and LDLC ≥190 mg/dl and/or personal or family history of premature cardiovascular disease and/or history of severe hypercholesterolemia. CVD was defined as carotid artery disease, history of stroke/TIA, coronary artery disease, congestive heart failure associated with CVD, and peripheral vascular disease.

Prior to initiation of therapy, all patients were counseled on a low cholesterol and saturated fat diet, and received follow-up counseling at serial visits. Instructions on how to use PCSK9 inhibitor auto-injector pens, education on its mechanism of action and side effects, and steps to be taken for missed doses were provided. Emergency contact information was given.

ALI and EVO were given in addition to patients’ entry maximal tolerated cholesterol lowering regimens. Insurance formulary coverage was taken into consideration when deciding whether to use ALI or EVO. ALI 75 mg was approved by insurance formulary coverage in 29 patients, 10 with entry LDLC ≥130 mg/dl, ALI 150 mg was approved for 18 patients, 15 with entry LDLC ≥130 mg/dl, and EVO 140 mg was approved in 22 patients, 17 with entry LDLC ≥130 mg/dl. Subcutaneous auto-injector pens were used every 2 weeks.

We previously [21] reported 24 week treatment follow-up for 23 of the 29 patients currently on ALI 75 mg, 12 of the 18 currently on ALI 150 mg, and 17 of the 22 currently on EVO 140 mg. Now we report extended follow-up for 29 patients on ALI 75 for a mean of 49 weeks, and for 40 on ALI-EVO for a mean of 37 weeks.

We recorded patient characteristics including age, gender, weight, body mass index, systolic and diastolic blood pressures, history of diabetes, smoking, and treatment with anti-hypertensive medications. Adverse events after the initiation of the therapy were recorded. Changes in 10-year cardiovascular risk were assessed using ACC/AHA [24] and NIH Framingham [25] risk calculators.

### Statistical methods

Statistical software SAS version 9.4 and Prism were used for data analysis and presentation.

To determine whether the ALI 150 mg and EVO 140 mg Q2W data could be pooled, stepwise multiple regression was carried out with absolute or percent change (from entry to last follow up) in LDLC as the dependent variable and age, BMI, LDLC at entry, sex, race, duration of follow-up, concomitant lipid lowering therapy, HeFH, CVD and three PCSK9 treatment groups as explanatory variables. The concomitant lipid lowering therapy was classified as statin alone, statin plus ezetimbe and/or colesevelam, only ezetimibe and/or colesevelam, and no lipid lowering therapy.

To determine if the LDLC lowering by ALI 75 mg or combined ALI-EVO was influenced by concomitant statin use, general linear models were used to calculate LS means of absolute or percent change in LDLC in patients taking statins, and in the no statin group after adjusting for PCSK9 treatment and treatment duration, age, BMI and LDLC at entry, race, gender, HeFH (+/−), and CVD (+/−).

Paired Wilcoxon tests were used to compare entry and follow-up data. Chi-square tests were used to assess the adverse effects (any vs none) between ALI 75 and combined ALI-EVO groups, and between taking statin and not taking statin groups.

## Results

Table 1 displays entry characteristics of our cohort of 69 patients. Median age at entry was 64 years, 88% Caucasian, 9% African-American, 1% Asian, and 1% Indian. Of the 69 patients, 52% were female, 48% male, 13% had diabetes, 4% smoked, and 62% were on anti-hypertensive medication. Of the 69 patients, 25 (36%) had HeFH only, 25 (36%) had CVD only, and 19 (28%) had both HeFH and CVD (Table 1). Of the 69 patients, 46 (67%) could not tolerate any dose of statin (Table 1). Before starting ALI or EVO, 13 patients were taking a statin only, 3 statin and ezetimibe, 1 statin and colesevelam, 6 statin, ezetimibe, and colesevelam, and 7 ezetimibe and/or colesevelam and 39 (57%) took no lipid lowering therapy (Table 1).

**Table 1:** 69 patients at study entry before treatment with Alirocumab or Evolocumab

Age at entry (years) Mean ± SD, [25th, 50th, 75th percentiles]	61.7 ± 9.4, [55, 64, 69]
BMI (kg/m^2^) Mean ± SD, [25th, 50th, 75th percentiles]	29.5 ± 4.8, [25.5, 29.0, 32.0]
Race	61 White (88%), 6 Black (9%), 1 Asian (1%), 1 Indian (1%)
Gender	36 F (52%), 33 M (48%)
Diabetes	9 Yes (13%), 60 No (87%)
Smoke	3 Yes (4%), 66 No (96%)
Antihypertensive drug	43 Yes (62%), 26 No (38%)
HeFH	44 Yes (64%), 25 No (36%); 25 had HeFH & no CVD (36%)
CVD	44 Yes (64%), 25 No (36%); 25 had CVD & no HeFH (36%)
Both HeFH & CVD	19 (28%)
Statin intolerant	46 Yes (67%), 23 No (33%)
Medication use at entry Taking Statin (*n* = 23)	Statin only, *N* = 13
	Statin + ezetimibe, *N* = 3
	Statin + colesevelam, *N* = 1
	Statin + ezetimibe + colesevelam, *N* = 6
Not taking statin (*n* = 46)	Ezetimibe only, *N* = 2
	Colesevelam only, *N* = 2
	Ezetimibe + colesevelam, *N* = 3
	Nothing, *N* = 39
Follow up weeks Mean ± SD, [25th, 50th, 75th %tiles] on ALI 75 mg Q2W (*n* = 29)	49 ± 13, [38, 49, 59]
on ALI 150 mg (*n* = 18) or EVO 140 mg (*n* = 22) Q2W, data pooled	37 ± 12 [26, 33, 45]
Total cohort (*n* = 69)	42 ± 14 [30, 40, 51]

By stepwise regression, there was no difference in LDLC lowering (*p* > 0.05) between ALI 150 mg and EVO 140 mg. Hence, we pooled data from 18 patients taking ALI 150 mg and 22 taking EVO 140 mg Q2W (ALI-EVO).

As displayed in Table 2, on ALI 75 mg, median LDLC fell from 115 mg/dl at entry to 68 mg/dl after a mean of 49 weeks treatment, a median 39% decrement, *p* < .0001. Ten year calculated CVD risk by the AHA calculator fell from 11.5 to 7.3% (*p* = .0008), a median decrement of 20% (*p* = .004), and by the NIH calculator fell from 12.9 to 5.1% (*p* < .0001), a median decrement of 67%, *p* < .0001, Table 2.

**Table 2:** Changes in LDLC and CVD risk from study entry to last follow up visit in 69 patients taking Alirocumab 75 mg every 2 weeks (*n* = 29), or Alirocumab 150 mg (*n* = 18) or Evolocumab 140 mg (*n* = 22) every 2 weeks (ALI-EVO)

		Alirocumab 75 mg every 2 weeks (*n* = 29) Mean ± SD, median follow-up length 49 ± 13, 49 weeks			ALI-EVO (*n* = 40) Mean ± SD, median 37 ± 12, 33 weeks		
Variable measured		percentile			percentile		
		25th	50th	75th	25th	50th	75th
LDLC	Entry (mg/dl)	99	115	143	136	165	196
	Follow up (mg/dl)	49	68	89	45	70	93
	Absolute change (mg/dl)	−26	−43	−81	−73	−91	−122
	P (paired Wilcoxon)	*p* < .0001			*p* < .0001		
	Percent change (%)	−22	−39	−62	−49	−59	-68
	P (Wilcoxon)	*p* < .0001			*p* < .0001		
CVD risk for next 10 years With AHA calculator	Entry (%)	4.8	11.5	18.1	3.9	10.2	19.4
	Follow up (%)	4.4	7.3	14.2	2.2	5.5	15.5
	Absolute change	−0.1	−1.2	−6.9	−0.6	−2.4	−4.8
	P (paired Wilcoxon)	*p* = .0008			*p* < .0001		
	Percent change (%)	−2.2	−19.9	−34.4	−12.8	−27.8	-50.5
	P (Wilcoxon)	*p* = .0042			*p* < .0001		
CVD risk for next 10 years With NIH calculator	Entry (%)	6.8	12.9	21.7	9.1	14.2	24.8
	Follow up (%)	2.1	5.1	8.3	1.4	3.6	8.5
	Absolute change	−4.3	−6.9	−16.9	−5.0	−10.1	−18.8
	P (paired Wilcoxon)	*p* < .0001			*p* < .0001		
	Percent change (%)	−52.2	−67.4	−80.4	−56.1	−77.8	-85.4
	P (Wilcoxon)	*p* < .0001			*p* < .0001		

On ALI-EVO for a mean of 37 weeks, median LDLC fell from 165 mg/dl at entry to 70 mg/dl, a median 59% decrement, *p* < .0001, Table 2. On ALI-EVO, 10-year calculated CVD risk by the AHA calculator fell from 10.2 to 5.5% (median decrement 28%, *p* < .0001), and by the NIH calculator from 14.2 to 3.6% (median decrement 78%, *p* < .0001), Table 2.

**Fig. 1:**
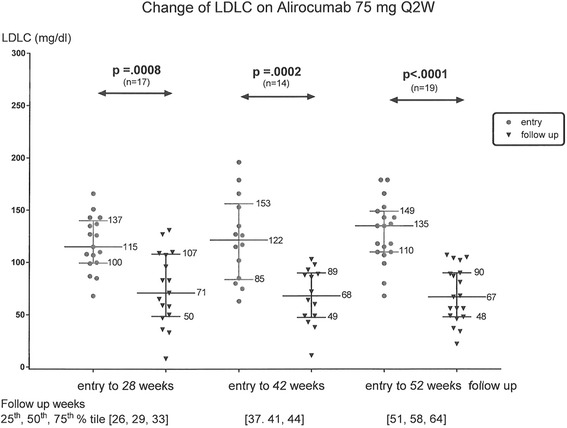
Change in LDLC from entry to 28, 42, and 52 weeks on Alirocumab 75 mg every 2 weeks in 29 patients; median, 25th and 75th LDLC percentiles displayed

Checking all LDLC measures during this extended follow up, in the ALI 75 mg group, median LDLC fell from 115 mg/dl at entry to 71 mg/dl at 28 weeks (*p* = .0008), from 122 mg/dl at entry to 68 mg/dl at 42 weeks (*p* = .0002), and from 135 to 67 mg/dl at 52 weeks (*p* < .0001), Fig. 1.

**Fig. 2:**
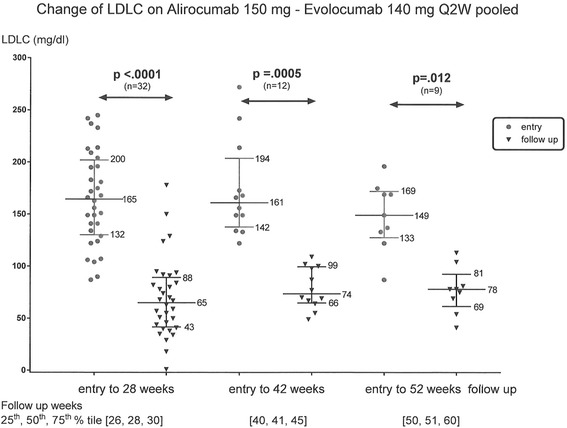
Change in LDLC from entry to 28, 42, and 52 weeks on Alirocumab 150 mg (*n* = 18) and Evolocumab 140 mg (*n* = 22) every 2 weeks (data pooled); median, 25th and 75th LDLC percentiles displayed

In the ALI-EVO group, median LDLC fell from 165 mg/dl at entry to 65 mg/dl at 28 weeks (*p* < .0001), from 161 mg/dl at entry to 74 mg/dl at 42 weeks (*p* = .0005), and from 149 to 78 mg/dl at 52 weeks (*p* = .012), Fig. 2.

On ALI 75 mg Q2W, 15 of 29 patients (52%) had ≥1 LDLC <70 mg/dl, and 58% of their LDLC measures were <70 mg/dl, Table 3. On ALI-EVO, 20 of 40 (50%) patients had ≥1 LDLC <70 mg/dl, and 47% of all of their LDLC determinations were <70 mg/dl, Table 3. For all 69 patients, 51% of patients had ≥1 LDLC <70 mg/dl on therapy, and 53% of all LDLC measures were <70 mg/dl, Table 3.

**Table 3:** Number (%) of patients who had at least one measure of LDLC <70 mg/dl, and number (%) of LDLC measurements of LDLC with LDLC <70 mg/dl through follow-up on ALI 75 mg every 2 weeks (*n* = 29), or Alirocumab 150 mg (*n* = 18) or Evolocumab 140 mg (*n* = 22) every 2 weeks (ALI-EVO)

	HeFH only, 25 patients	CVD only, 25 patients	HeFH & CVD, 19 patients	Total cohort, 69 patients
	Entry LDLC 25th, 50th, 75th percentile [141, 156, 181 mg/dl]	Entry LDLC 25th, 50th, 75th percentile [90, 110, 149 mg/dl]	Entry LDLC 25th, 50th, 75th percentile [106, 156, 204 mg/dl]	Entry LDLC 25th, 50th, 75th percentile [110, 143, 172 mg/dl]
	number (%) of patients had LDLC < 70 mg/dl at least once during follow up, number (%) of measures with LDLC <70 mg/dl during follow up			
ALI 75 mg/2 weeks Total 29 patients 65 LDLC measurements	2/6 (33%) patients 8/17 (47%) LDLC measurements	11/17 (65%) patients 22/35 (63%) LDLC measurements	2/6 (33%) patients 8/13 (62%) LDLC measurements	15/29 (52%) patients 38/65 (58%) LDLC measurements
ALI-EVO Total 40 patients 59 LDLC measurements	9/19 (47%) patients 12/27 (44%) LDLC measurements	6/8 (75%) patients 9/12 (75%) LDLC measurements	5/13 (38%) patients 7/20 (35%) LDLC measurements	20/40 (50%) patients 28/59 (47%) LDLC measurements
All treatment groups Total 69 patients 124 LDLC measurements	11/25 (44%) patients 20/44 (45%) LDLC measurements	17/25 (68%) patients 31/47 (66%) LDLC measurements	7/19 (37%) patients 15/33 (45%) LDLC measurements	35/69 (51%) patients 66/124 (53%) LDLC measurements

On ALI 75 mg, median TG fell from 130 to 124, *p* = 0.06, and median HDLC was unchanged, Table 4. Median total cholesterol fell from 192 to 152, a median 26% reduction, *p* < .0001. Median non-HDL cholesterol fell from 136 to 50, a median 54% reduction, *p* < .0001, Table 4.

**Table 4:** Change in total cholesterol, triglyceride, HDL cholesterol, and Non-HDL cholesterol in 69 patients treated with Alirocumab 75 mg every 2 weeks (*n* = 29), or Alirocumab 150 mg (*n* = 18) or Evolocumab 140 mg (*n* = 22) every 2 weeks (ALI-EVO)

		Alirocumab 75 mg every 2 weeks (*n* = 29) Mean ± SD, median follow-up length 49 ± 13, 49 weeks			ALI-EVO (*n* = 40) Mean ± SD, median 37 ± 12, 33 weeks		
Variable measured		percentile			percentile		
		25th	50th	75th	25th	50th	75th
Total cholesterol	Entry (mg/dl)	172	192	230	224	255	288
	Follow up (mg/dl)	127	152	173	124	154	180
	Absolute change (mg/dl)	−14	−54	−95	−71	−105	−132
	P (paired Wilcoxon)	*p* < .0001			*p* < .0001		
	Percent change (%)	−6	−26	−38	−35	−39	-49
	P (Wilcoxon)	*p* < .0001			*p* < .0001		
Triglyceride	Entry (mg/dl)	96	130	184	129	161	233
	Follow up (mg/dl)	81	124	150	90	119	167
	Absolute change (mg/dl)	+7	−12	−53	−1	−47	−102
	P (paired Wilcoxon)	*p* = .060			*p* < .0001		
	Percent change (%)	+6	−15	−29	−0.3	−32	-43
	P (Wilcoxon)	*p* = .082			*p* < .0001		
HDL cholesterol	Entry (mg/dl)	40	51	61	42	50	58
	Follow up (mg/dl)	41	51	65	49	55	63
	Absolute change (mg/dl)	−4	0	+7	−1	+5	+10
	P (paired Wilcoxon)	*p* = .40			*p* = .0008		
	Percent change (%)	−9	0	+12	−2	+8	+21
	P (Wilcoxon)	*p* = .41			*p* = .0002		
Non-HDL cholesterol	Entry (mg/dl)	122	136	181	170	201	232
	Follow up (mg/dl)	25	50	75	70	92	121
	Absolute change (mg/dl)	−27	−54	−94	−82	−112	−131
	P (paired Wilcoxon)	*p* < .0001			*p* < .0001		
	Percent change (%)	−17	−37	−54	−46	−54	-62
	P (Wilcoxon)	*p* < .0001			*p* < .0001		

For the ALI-EVO group, median TG fell from 161 to 119, a median 32% reduction, *p* < .0001, and HDLC rose from 50 to 55 mg/dl, a median 8% increase, *p* = .0002, Table 4. Total cholesterol fell from 255 to 154, a 39% reduction, *p* < .0001. Median non-HDL cholesterol fell from 201 to 92 mg/dl, a median 54% reduction, *p* < .0001, Table 4.

Neither absolute nor percentage reduction of LDLC differed between patients taking or not taking statins at entry and throughout the study, Table 5.

**Table 5:** Comparisons of LDLC change between statin tolerant and intolerant groups

	Statin tolerant (*n* = 23)	Statin intolerant (*n* = 46)
LS means^1^ ± SE of absolute change in LDLC (mg/dl)	−86 ± 8	−75 ± 5
Group differences	*p* = .21	
LS means^1^ ± SE of percentage change in LDLC (%)	-58 ± 7	−48 ± 4
Group differences	*p* = .19	

^1^Least Square means for statin taking and not taking groups, adjusted for PCSK9 treatment (2 groups), treatment duration, age, BMI, race, gender, HeFH (yes-no), CVD (yes-no) and LDLC at entry.

The most common side effect overall was flu-like symptoms in 14% of patients, followed by fatigue (7%) in the ALI 75 mg group and by headache or gastrointestinal symptoms (5% for each) in the ALI-EVO group, Table 6. No patients reported reduced cognitive function. Side effects did not differ (*p* = 0.11) between ALI 75 and ALI-EVO groups, Table 6.

**Table 6:** Adverse events in 69 patients. 29 on Alirocumab 75 mg every 2 weeks, and Alirocumab 150 mg (*n* = 18) or Evolocumab 140 mg (*n* = 22) every 2 weeks (ALI-EVO)

	All treatment groups (*n* = 69)	Alirocumab 75 mg every 2 weeks (*n* = 29)	ALI-EVO (*n* = 40)
	Mean ± SD, median follow-up length	Mean ± SD, median follow-up length	Mean ± SD, median follow-up length
	42 ± 14, 40 weeks	49 ± 13, 49 weeks	37 ± 12, 33 weeks
Flu-like symptoms	10 (14%)	1 (3%)	9 (23%)
Respiratory tract infection /symptoms	2 (3%)	1 (3%)	1 (3%)
Inject site reaction	1 (1%)	1 (3%)	
Fatigue	2 (3%)	2 (7%)	
Headache	2 (3%)		2 (5%)
Urticaria /itchiness	2 (3%)	1 (3%)	1 (3%)
G.I. symptom	2 (3%)		2 (5%)
Weight gain	2 (3%)	1 (3%)	1 (3%)
Hair loss	1 (1%)		1 (3%)
Any adverse events	24 (35%)	7 (24%)	17 (43%)

Compare adverse events (any events) in the 2 treatment groups, χ^2^ = 2.50, *p* = .11

When separating the cohort by entry statin intolerance, the number of adverse events experienced during ALI and EVO therapy did not differ (*p* = 0.11), Table 7.

**Table 7:** Adverse events in 69 patients on Alirocumab 75 mg or Alirocumab 150 mg or Evolocumab 140^1^ mg every 2 weeks, by entry statin intolerance group

	Statin tolerant, taking statin (*n* = 23)	Statin intolerant (*n* = 46)
	Follow up length	Follow up length
	25th, 50th, 75th %tile	25th, 50th, 75th %tile
	28, 39, 16 weeks	30, 40, 52 weeks
Flu-like symptoms	2 (9%)	8 (17%)
Respiratory tract infection /symptoms	1 (4%)	1 (2%)
Inject site reaction	1 (4%)	
Fatigue	0	2 (4%)
Headache	0	2 (4%)
Urticaria /itchiness	0	2 (4%)
G.I. symptom	0	2 (4%)
Weight gain	1 (4%)	1 (2%)
Hair loss	0	1 (4%)
Any adverse events	5 (22%)	19 (41%)

Compare adverse events (any events) in the 2 groups, χ^2^ = 2.59, *p* = .11

One patient had coronary bypass revision due to scar tissue growth within 1 month of starting ALI 150 mg Q2W. The ALI 150 mg was not stopped and the event was not attributed to the ALI therapy.

## Discussion

Despite maximal tolerated cholesterol lowering therapy, many patients fail to achieve optimal LDLC lowering [26–28], with only 28% of patients in NHANES achieving LDLC <70 mg/dl on treatment. [29] Failure to reach optimal LDLC lowering is predominantly related to statin intolerance [30–34]. In the current study, 67% of patients were statin intolerant, a very common, outcome limiting problem in treatment of hypercholesterolemia [22, 23, 32–35]. Of patients who discontinue statins, 60% report statin intolerance as the reason [36]. In the current study, where 67% of patients were statin intolerant at entry, reduction of LDLC by ALI or ALI-EVO did not differ between patients with or without entry statin intolerance. LDLC lowering and tolerability of EVO in the current study was congruent with evaluation of patients with statin intolerance in GAUSS-3, where EVO was well-tolerated and effective [1]. PCSK9 inhibitors now offer the promise of optimizing LDLC in the majority of patients with HeFH, CVD, and concurrent statin intolerance as previously published [2–5, 37–39], and as in the high risk HeFH-CVD patients of current report, 67% of whom could not take any statin at any dose or sequence.

ALI and EVO have been found to be very efficacious and safe during phase II and III randomized controlled trials with minimal adverse events compared to placebo [1, 2, 4, 40, 41]. During phase III trials with patients on maximal tolerated cholesterol lowering therapy along with ALI 150 mg and ALI 75 mg Q2W, there were 61% and 46% reductions from baseline in LDLC, respectively, at median 24 weeks [2, 11]. In OSLER-1 and 2, patients on EVO 140 mg Q2W or 420 mg once/month had LDLC reduction by 61% at median 12 weeks on top of antecedent cholesterol lowering therapy [4].

Meeting both FDA indications and third party insurance drug coverage requirements, our current study was done in HeFH and/or CVD patients with suboptimal cholesterol lowering despite maximal tolerated cholesterol lowering therapy. This qualified all of our cohort, with minimal exclusion criteria, for initiation of ALI or EVO therapy, a cohort much more diverse than those in the placebo-controlled randomized clinical trials [2, 4]. Although statin therapy is successful in reducing CVD events, it has suboptimal success in cohorts enriched with HeFH [42]. Over a 9-year follow-up period, 39% of 255 patients studied by Rallidis et al. had a major adverse coronary event despite 84.3% being on statins, with only 2.3% achieving LDLC <70 mg/dl [42]. Our current study cohort included 25 patients with HeFH only, 25 with CVD only, and 19 with both. Moreover, in our current study, on ALI 75 mg Q2W, 52% of patients had ≥1 LDLC <70 mg/dl, and LDLC was <70 mg/dl in 58% of their measures. On ALI-EVO, 50% of patients had ≥1 LDLC <70 mg/dl, and 47% of their LDLC determinations were <70 mg/dl.

LDLC reduction in current study for ALI-EVO (59%) was within 2–3% of that reported in the placebo-controlled trials for ALI 150 mg (62%)^2^ or EVO 140 mg (61%)^4^ while in the ALI 75 mg group, median LDLC lowering was 39% compared to 48.9% in the Odyssey Phase 3 clinical trials [43]. In our cohort where 67% of patients were statin intolerant, ALI 75 mg and ALI-EVO therapy was successful in high-risk patients with HeFH and/or CVD who otherwise could not achieve LDLC <70 mg/dl with maximal tolerated LDLC lowering regimens.

In our current study, median LDLC was reduced to 68 mg/dl by ALI 75 and to 70 mg/dl by ALI-EVO, with 39% and 59% reduction from entry on best tolerated LDLC lowering. The median absolute reduction of LDLC on ALI 75 was 43 mg/dl, and on ALI-EVO 91 mg/dl. Reduction of LDLC by 77 mg/dl for 5 years in 10,000 patients has been estimated to prevent major vascular events in 1000 (10%), an absolute benefit in those who had pre-existing CVD, and in 500 patients (5% absolute benefit) in primary prevention [44].

From past vascular studies on statins, regression of plaque can be induced when LDLC is held ~70 mg/dl or below [45, 46]. When patients were given rosuvastatin 40 mg in the ASTEROID trial, mean LDLC was reduced from 130 mg/dl to 60 mg/dl (53%), with a total atheroma volume reduction of 6.8% as well as a significant reduction in all intravenous ultrasound measurements of atheroma burden [47]. Consequently, in the recent GLAGOV study [20], compared with statin-placebo, the EVO-statin group achieved lower mean LDLC (36.6 vs 93 mg/dL, *p* < .001). The primary efficacy parameter, percent atheroma volume (PAV), increased 0.05% with placebo and decreased 0.95% with EVO, *p* < .001). EVO induced plaque regression in more patients than placebo (64.3% vs 47.3%, *p* < .001 for PAV, and 61.5% vs 48.9%, *p* < .001 for total atheroma volume (TAV). The GLAGOV study also demonstrated a positive linear change in percent PAV as LDLC increased from 20 mg/dl to 110 mg/dl [20].

It has been estimated that a lifetime reduction of LDLC ~40 mg/dl would reduce risk of CVD by 50% [48]. In our current study, the median absolute LDLC reduction ranged from 43 mg/dl (ALI 75 mg) to 91 mg/dl (ALI-EVO), and 53% of all LDLC measures on therapy were <70 mg/dl. Moreover, according to the AHA and NIH 10-year CVD risk calculators, on ALI 75 mg there was CVD risk reduction of 20% and 67% respectively. On ALI-EVO, by the AHA and NIH calculators, there were 28% and 78% reductions estimated in 10-year CVD risk. The ACC/AHA calculator was not, however, designed for use in patients with pre-existing CVD events, although the NIH calculator has no such restriction [49]. In at least 50% of our patients on PCSK9 therapy with LDLC < 70 mg/dl, from the past experience with vascular studies on statins [45–47] and recent GLAGOV [20] study, we speculate that there should be significant regression of vascular plaque.

In the current study, both ALI and EVO were generally well-tolerated; the most significant frequent adverse event was flu-like myositis-myalgia in 14% of patients overall. There were, however, no differences between groups (ALI 75 vs ALI-EVO) in adverse events (*p* = 0.11). This is comparable to the pattern of side effects for ALI and EVO in randomized placebo-controlled trials [50, 51]. In a meta-analysis of 25 randomized controlled trials with PCSK9 inhibitors, there were no significant differences in major adverse event rates between the active drug and control treatment [52].

A strength of our current report is the extension of post-commercialization follow-up of LDLC lowering and safety in a high risk population from 24 weeks to a mean of 37 weeks for the pooled ALI-EVO group, and to a mean of 49 weeks for patients receiving ALI 75 mg Q2W. A limitation of this study is the relatively small group of patients. A second limitation is a probable bias towards higher risk patients with HeFH, CVD, and statin intolerance, unable to reach LDLC lowering goals on conventional LDLC-lowering therapy, by virtue of referral to a regional cholesterol treatment center.

## Conclusions

In hypercholesterolemic patients with HeFH, and/or CVD with suboptimal LDLC lowering on maximal tolerated LDLC-lowering therapy at entry, LDLC was reduced 39% on ALI 75 mg from 115 to a median of 68 mg/dl, and by 59% on ALI-EVO from 165 to a median of 70 mg/dl. Reported adverse events were minimal and tolerable. ALI and EVO represent paradigm shifts in LDLC lowering.

## Abbreviations

AHA: American Heart Association
ALI: Alirocumab
ASCVD: Atherosclerotic Cardiovascular Disease
CVD: Cardiovascular Disease
EVO: Evolocumab
HeFH: Heterozygous familial hypercholesterolemia
LDLC: Low-Density Lipoprotein Cholesterol
MI: Myocardial Infarction
NIH: National Institutes of Health
PAV: Percent Atheroma Volume
PCSK9: Proprotein convertase subtilisin/kexin type 9
Q2W: Every 2 weeks
Q4W: Every 4 weeks
TAV: Total Atheroma Volume
TEAE: Treatment Emergent Adverse Effect
TIA: Transient Ischemic Attack
